# Supplementary material for: Vitamin D Modified DSS-Induced Colitis in Mice via STING Signaling Pathway
Source: Biology (Basel). 2025 Jun 18;14(6):715. doi: 10.3390/biology14060715 (PMC12190092; doi:10.3390/biology14060715)
Supplement: Supplementary file 1 [file biology-14-00715-s001.zip › Supplementary Materials S1: 16s-report of gut microbiota/Groups/diff_analysis/RF/Bugbase_Groups.RFroc.pdf]

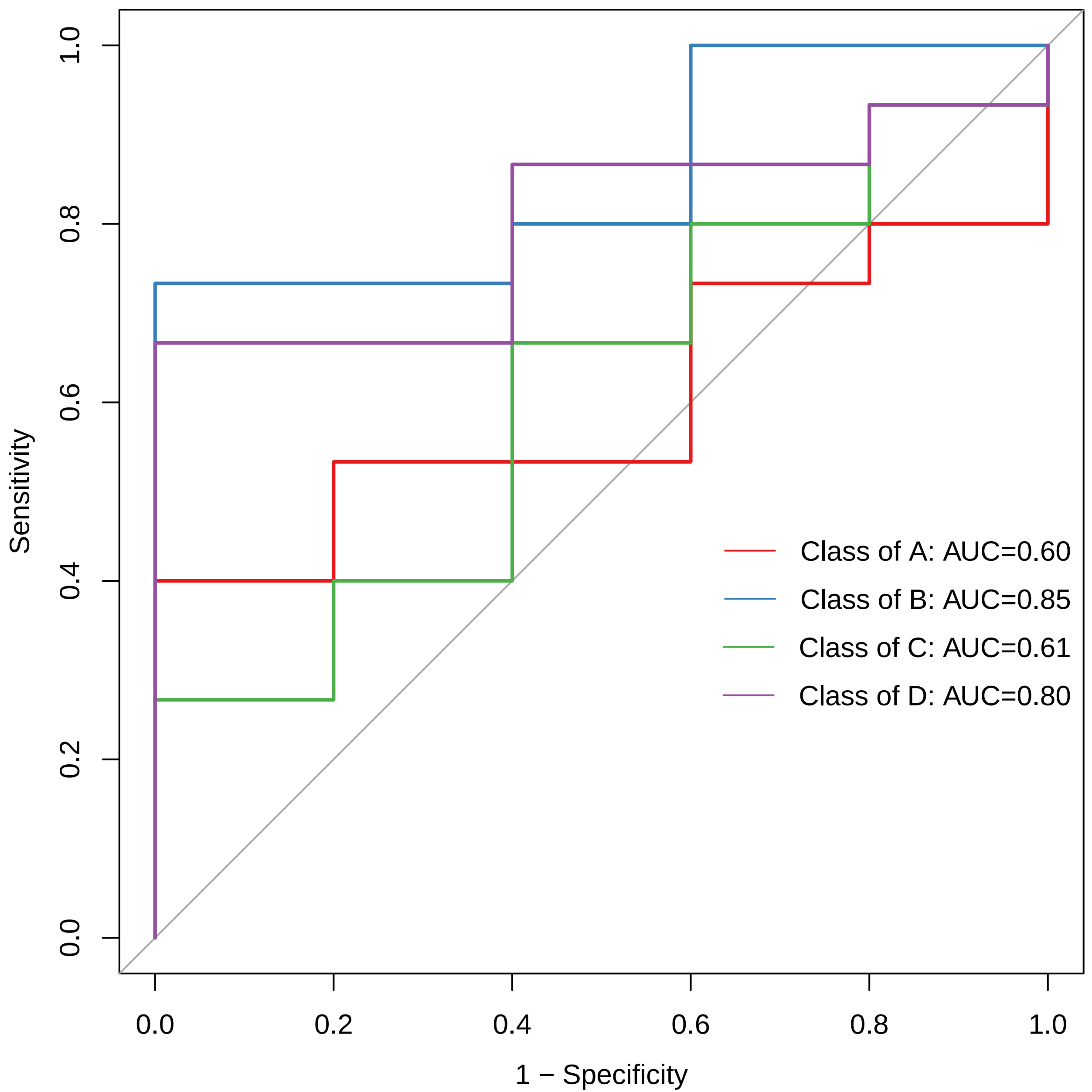
Class of A: AUC=0.60
Class of B: AUC=0.85
Class of C: AUC=0.61
Class of D: AUC=0.80
Sensitivity
1 − Specificity
0.0
0.2
0.4
0.6
0.8
1.0
